# Hexaaqua­copper(II) dinitrate: absence of Jahn–Teller distortion. Corrigendum

**DOI:** 10.1107/S1600536812049732

**Published:** 2012-12-08

**Authors:** Ramin Zibaseresht, Richard M. Hartshorn

**Affiliations:** a Department of Chemistry, University of Canterbury, Private Bag 4800, Christchurch, New Zealand

## Abstract

The identity of the metal atom in the paper by Zibaseresht & Hartshorn [*Acta Cryst.* (2006), E**62**, i19–i22] is corrected.

## Abstract 2

Corrigendum to *Acta Cryst.* (2006), E**62**, i19–i22.

The published crystal structure in the paper by Zibaseresht & Hartshorn (2006) ▶ is almost certainly that of the nickel complex and not the copper complex as originally identified. Refinement of the copper occupancy was consistent with the presence of a lighter transition metal ion in the structure, and residuals improved significantly when the complex was refined as the nickel or cobalt salts. Unfortunately the original samples are no longer available, so further analysis of the crystals is not possible, but based on the colour of the crystals (blue) we believe the metal to be nickel.
